# Oral Health Problems and Utilization of Dental Services among Spanish and Immigrant Children and Adolescents

**DOI:** 10.3390/ijerph17030738

**Published:** 2020-01-23

**Authors:** Silvia Portero de la Cruz, Jesús Cebrino

**Affiliations:** 1 Department of Nursing, Pharmacology and Physiotherapy, Faculty of Medicine and Nursing, University of Córdoba, Avda. Menéndez Pidal, S/N, 14071 Córdoba, Spain; 2 Department of Preventive Medicine and Public Health, Faculty of Medicine, University of Seville, Avda. Doctor Fedriani, S/N, 41009 Seville, Spain; jcebrino@us.es

**Keywords:** adolescent, child, dental health services, emigrants and immigrants, oral health, Spain

## Abstract

Spanish and immigrant children and adolescents vary widely in their frequency of dental visits and occurrence of dental problems. The aims of this study were to discover the prevalence of dental problems and utilization of dental services in the Spanish and immigrant child population, identify the type of treatment received, and analyze the socioeconomic and demographic variables which are associated with dental problems and non-regular utilization of dental services, based on data from the 2017 National Health Survey in Spain. The sample consisted of 4568 children aged between 3 and 14 years old. Utilization of dental services and dental problems were assessed against socioeconomic and demographic characteristics using logistic regression models. The prevalence of caries in Spanish children was 9.29% compared with 18.58% (*p* < 0.001) in their immigrant counterparts. The most common reason for dental visits was a check-up (Spanish: 65.05%; immigrants: 54.94%). In both groups, from the age of 7, there was a lower probability of non-regular utilization of dental services, although this increased when the social class was lower. The probability of presenting dental problems was lower in Spanish children living in towns with over 10,000 inhabitants and was higher, in both groups, over the age of 7 and in lower social classes.

## 1. Introduction

Dental health problems include a wide range of chronic clinical conditions, from tooth decay or periodontal disease to oral cancers. Despite the fact that these conditions are largely preventable, they still affect over 3.5 billion people worldwide [1].

Regular visits to a dental clinic help to prevent, identify and treat dental health problems, thus improving the quality of life for both individuals and society as a whole [2]. In Spain, although the public health service offers free dental care for children up to the age of 16, except for orthodontic services [3], dental health care is mainly provided through the private system and each visit is paid for. However, the frequency of utilization of dental services seems to be uneven among the child-adolescent population [4], depending on the parents’ educational and socioeconomic level, beliefs about dental health, perception of current dental health status, age, and gender [5]. In addition, it has been shown that in Spain, the European country with the fourth highest percentage of immigrants in the population (9.72%) [6], the frequency of utilization of dental services is lower among immigrant children compared with Spanish children [7]. This group is in a disadvantaged social and economic position, and is therefore more vulnerable to problems of dental health and access to dental health services [8].

In addition, the study of oral diseases (mainly dental caries), reveals worse levels of dental health in immigrant or migrant children [8]. Although there is no consensus about its impact, it has been hypothesized that socioeconomic status predicts or even casually affects oral disease [9]. A recent systematic review has shown that a low socioeconomic level is significantly associated with high risk of tooth decay [10]. On the other hand, a study assessing dental health in 6–17-year-olds revealed that tooth decay was more frequent in children with a moderate-high socioeconomic level [11]. Although the influence of number of demographic factors has been considered, such as the children’s age, gender, or place of residence, the findings are noticeably contradictory [12].

In view of the fact that dental diseases involve major economic costs to society, both directly (costs of treatment) and indirectly (e.g., poorer academic performance) [13], and that there seem to be inequalities in access to dental treatment and in the presence of dental problems according to the children’s country of origin or ethnicity [7], there is a clear need to study the socioeconomic and demographic factors involved, so that prompt, efficient action can be taken to reduce these inequalities in health provision [14]. The main objectives of the present study were therefore: (i) to discover how frequently dental services are used and the prevalence of dental health problems in Spanish and immigrant children; (ii) to identify the type of treatment received during dental health visits; and (iii) to analyze the socioeconomic and demographic variables which influence the non-regular utilization of dental health services and the presence of dental health problems.

## 2. Materials and Methods

A cross-sectional study was carried out from September to December 2019 using the data obtained from the 2017 Spanish National Health Survey (SNHS) [15]. The SNHS is a representative survey of the general population (representativeness is ensured by assigning a weighting coefficient to each participant) carried out by the Ministry of Health, Consumption and Social Welfare in partnership with the National Institute of Statistics. The data for the SNHS 2017 were collected over the period of a year, from October 2016 to October 2017. Trietapic sampling was used, stratified by census areas, family homes and individuals. The data analyzed on the individual level were obtained from the Children’s Questionnaire (0–15 years) and the Household Questionnaire. The questionnaires are usually filled in by the mother, father or guardian, who generally know most about all aspects related to the child’s state of health and health care. The analysis included children aged 3–14, as these were the target population of children’s oral health plans. Children with dual nationality were excluded from the study. The initial sample consisted of 4957 subjects, but due to a lack of data for some of the variables studied, 389 (7.85%) were excluded when the bivariate and multivariate statistical analyses were carried out (research data can be found in Supplementary Materials).

The dependent variables were: (i) utilization of dental services; and (ii) dental health problems. The analysis of the variable “*utilization of dental services*” was based on the question about “*time since the last visit to the dentist, stomatologist or dental hygienist*”, defined as regular utilization (visit in last twelve months or less) or irregular utilization (last visit more than twelve months ago), following institutional recommendations for check-ups on at least a once-yearly basis [16]. The “*dental health problems*” variable was assessed through the questions “*Do you have caries?*”, “*Do you have any teeth/molars with fillings or capped teeth?*” and “*Do your gums bleed spontaneously or when you brush your teeth?*” Answering yes to at least one of these questions confirmed the existence of dental health problems.

The independent variables were: gender (male/female); age, categorized according to increasing levels of autonomy from caregiver (3–6/7–10/11–14 years old); the child’s nationality, where the subjects were classified as immigrants if they answered “*yes*” to the question “*Foreign nationality?*” and those who answered “*yes*” to the question “*Spanish nationality?*” were classified as Spanish; size of town of residence, divided into three categories (<10,000 inhabitants/10,000–100,000 inhabitants/>100,000 inhabitants); type of household (couple with children under 25/father or mother only with children under 25/other type of household). In addition, social class was obtained from the current or former occupation of the head of the household and based on the nine categories proposed by the Classifications Working Group of the Spanish Society of Epidemiology [17]. In the SNHS 2017, occupation is divided into six categories: Class I (directors and managers of companies with 10 or more employees and professionals normally qualified with university degrees); Class II (directors and managers of companies with less than 10 salaried employees, professionals normally qualified with university degrees, other technical support professionals, athletes, and artists), Class III (intermediate professions and self-employed workers); Class IV (supervisors and workers in skilled technical work); Class V (skilled workers in the primary sector and other semi-skilled workers); and Class VI (unskilled workers). In our study, we reorganized these groups into the following categories: upper class (Classes I and II); middle class (Classes III and IV); and lower class (Classes V and VI). Finally, the type of treatment received at the dentist, stomatologist or dental hygienist (check-up/dental cleaning/fillings, endodontic treatment, sealed cavities/tooth extraction/dental crowns, dental bridges or other type of prostheses/gum disease treatment/orthodontic pieces/application of fluoride/other treatments) was used as an independent variable. 

According to the SNHS methodology, the microdata files are anonymous and made available to the public. According to Spanish legislation, when secondary data is used, no approval is required from an accredited ethics committee.

The qualitative variables were obtained by calculating the counts and percentages and the quantitative variables by calculating the arithmetic mean and standard deviation (SD). The proportions of the categorical variables were compared using the chi-square test for contingency tables or Fisher’s exact test if the number of expected frequencies was greater than 5. Multivariate analyses were stratified by the children’s nationality to explore the relationship between socioeconomic and demographic characteristics and the presence of dental health problems (binary logistic regression with “*presence of dental health problems*” as the reference category) and with the non-regular utilization of dental services (binary logistic regression “*has not used dental services for more than twelve months*” as the reference category). Raw and adjusted odds ratios (ORs) were obtained for all the socioeconomic and demographic characteristics, with 95% confidence intervals. The Wald statistic was used to exclude one by one from the model any variables with a *p* ≥ 0.15 (backward methodical selection procedure). All the hypothesis contrasts were bilateral and in all the statistical tests, those with a 95% confidence level (*p* < 0.05) were considered significant values. The statistical analysis was carried out using the G-Stat program, version 2.0 (GlaxoSmithKline S. A., Madrid, Spain)

## 3. Results

### 3.1. Socio-Economic and Demographic Variables 

The final sample consisted of 4568 records of children between 3 and 14 years old. 94.46% (*n* = 4315) were Spanish and 5.54% (*n* = 253) immigrants. The average age of the Spanish children was 8.92 (SD = 3.46) years and that of the immigrants was 8.63 (SD = 3.45) years. In both groups, the majority were boys (Spanish: 51.24%; immigrants: 53.75%), who lived in towns with a population of 10,000–100,000 inhabitants (Spanish: 41.51%; immigrants: 67.98%), with both parents (Spanish: 77.27%; immigrants: 77.87%) and belonging to the lower social class (Spanish: 42.73%; immigrants: 71.14%).

### 3.2. Utilization of Dental Services and Oral Health Problems

As can be seen in Table 1, the utilization of dental services and oral health status differs between Spanish and immigrant children. The percentage of immigrant children who had not used dental services for over a year (51.78%) was higher than that of Spanish children (35.43%) (*p* < 0.001). In the latter group, a lower prevalence of caries (9.29%) was observed compared with the immigrant group (18.58%) (*p* < 0.001).

### 3.3. Type of Dental Treatment Received

**Figure 1:**
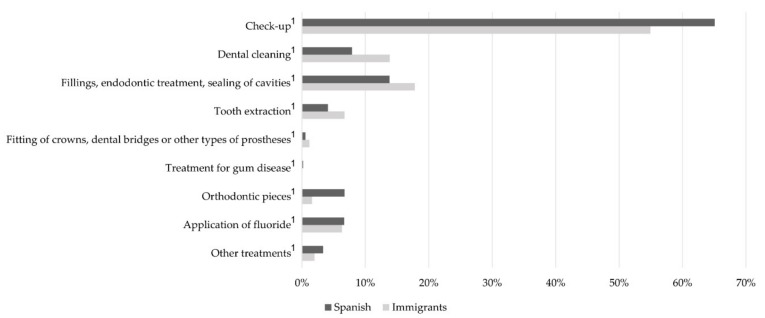
Type of assistance received at the dentist, stomatologist or dental hygienist for the population of Spanish and immigrant children and adolescents. Spanish National Health Survey, 2017. The graph shows the Spanish and immigrant child population (black and grey segments, respectively). ^1^ *p* < 0.001 by Fisher’s exact test.

Regarding the type of treatment received at the dentist (Figure 1), a check-up was the most common reason for visits in both groups (Spanish: 65.05%; immigrants: 54.94%). Dental cleaning (13.83%), fillings, endodontic treatment, sealing of cavities (17.79%), tooth extraction (6.72%), and the fitting of crowns, dental bridges or other types of prostheses (1.19%) were commoner in immigrant children than in their Spanish counterparts (*p* < 0.001).

### 3.4. Association between Socioeconomic and Demographic Variables and Utilization of Dental Services

The overall prevalence of non-regular utilization of dental services is distributed differently according to the socioeconomic and demographic variables (Table 2). The bivariate analysis reveals a higher prevalence of non-regular utilization in 3–6-year-olds (Spanish: 53.11%, *p* < 0.001; immigrants: 45.04%, *p* < 0.001) and in those belonging to the lower social class (Spanish: 47.22%, *p* < 0.001; immigrants: 77.86%, *p* = 0.04). In the multivariate analysis, in both groups, a clear upward trend was observed in the probability of non-regular utilization as the social class decreased, although this trend was inverted as the age of the children increased.

### 3.5. Association between Socioeconomic and Demographic Variables and the Presence of Dental Health Problems

Regarding the presence of dental health problems, the overall prevalence in Spanish children was 31.10% and 36.36% in immigrant children (Table 3). The bivariate analysis revealed, in both groups, a higher prevalence of dental health problems in 11–14-year-olds (Spanish: 48.58%, *p* < 0.001; immigrants: 47.83%, *p* < 0.001), and in Spanish children belonging to the most disadvantaged social class (45.83%, *p* = 0.02). After adjusting the model, the differences between the reference categories remained significant in all cases. Thus, from the age of 7, the probability of having dental health problems was more than double, in both groups, than at earlier ages, and this probability was even greater in children belonging to the lower social class, while the probability for Spanish children living in towns with > 10,000 inhabitants was lower than for those living in smaller towns.

## 4. Discussion

The prevalence of non-regular utilization of dental services in 3–14-year-old Spanish and immigrant children was high (Spanish: 35.43%; immigrants: 51.78%), and well above the current recommendations for regular once-yearly check-ups [16]. This disparity may be due to cultural differences in the way families seek dental health care, or problems with communication among the immigrant population [18,19]. Incidentally, it should be noted that immigrants currently have all the same rights to healthcare as the Spanish population [20].

The most common reason for dental visits among children was for a check-up; these findings were in line another study [21]. However, there were differences in the other reasons for visiting the dentist between Spanish and immigrant children. As found in another study [22], mouth cleaning, fillings, endodontic treatment, sealing cavities and dental extractions were commoner among immigrant children and treatment for gum disease, orthodontic treatment or the application of fluoride were more frequent among Spanish children.

Despite the increased access to dental health treatment for children and adolescents, social inequalities still remain in the frequency of use. For instance, a lower frequency of utilization was recorded in children belonging to the most disadvantaged social class, arguably due to a greater lack of information about health benefits [14]. Similarly, the age of the children significantly affected their utilization of the dental service. As found by Reda et al. [5], older subjects had a lower probability of non-regular utilization than 3–6-year-olds. This may reflect the lesser importance given by parents to early dental visits, especially with a view to looking after their primary teeth, which demonstrates the need to motivate parents to encourage their children to look after their teeth from the very earliest age.

Although a study of Mexican migrants in California found that girls were more likely to use dental services more frequently [23], this was not evident in the present study. Regarding the size of town, we found that the probability of children going over a year without visiting the dentist was comparable in both groups; similar results were found in another study [24]. However, the existence of differences in the utilization of dental services between the inhabitants of urban and rural areas has only been found in countries with a high Human Development Index (HDI) and could be the result of a combination of an unequal distribution of dental services, geographical distance and the shortage of means of transport in rural areas [7]. In addition, the type of household in which the children lived was not associated with a lower frequency of utilization of dental services. However, there was a link between family structure and the pattern of dental care. According to a recent systematic review and meta-analysis, children belonging to single-parent families used dental services less, regardless of the country’s HDI, compared with conventional nuclear families [5]. When only one parent is present on a regular basis, that parent often has less regular interaction and participation in the child’s daily activities, which makes it more difficult for them to schedule a visit to the dentist. On the other hand, this situation may give the child more opportunities to become more resilient or motivated to succeed [25].

Also, in line with another study [26], no relationship was found between the presence of dental health problems and the children’s gender. Most studies point to a higher prevalence of tooth decay in girls than in boys, as their permanent teeth appear earlier (thus leading to an earlier exposure to risk factors for dental illness), as well as differences in salivary composition or hormonal fluctuations during puberty. On the other hand, the findings from a number of studies have suggested that, except during puberty, women are less prone to severe periodontitis than men due to hormonal differences and better oral health habits [9,12,27]. Although children living in single-parent families are more likely to pick up poor habits of oral hygiene and have higher rates of caries in early childhood [28], this study also found no links between family structure and the existence of dental health problems. This is in line with the findings from other studies [29,30] and may be because the predominant age group in this study was 11 to 14 years. Children at this age, compared with younger children, have been able to develop their own oral hygiene habits and their behavior may not require such close parental supervision.

In both groups, from the age of 7, the likelihood of suffering from dental health problems increased. This result may be explained by the change in dental health habits during late childhood and early adolescence: while parents often check that young children brush their teeth, adolescents brush their teeth with varying degrees of proficiency and commitment. Other factors to take into consideration at this stage include the increased influence of the peer group and the media, and the greater availability and consumption of sugary food and drinks [31,32]. The results of this study are in line with other studies carried out both nationally [33] and internationally [28], which show that belonging to the most disadvantaged social class is closely linked to the presence of dental problems in children and adults. This implies that prevention strategies should be changed and aimed specifically at these risk groups. On the other hand, Spanish children who lived in areas with a higher population had a lower probability of suffering from dental problems. This situation, similar to that found in some studies [12,34] and contrary to others [9,35], may be due to the fact that in rural areas, children live further away from, and so have more limited access to, shops, kiosks and fast food restaurants. This can contribute to lower sugar intake and fewer snacks between meals. However, in one particular area, dental health status may vary between subgroups [12], which shows that further studies are required to explore dental health in relation to sociodemographic characteristics.

The results of this study should be taken into consideration by health authorities when designing (or improving current) dental health programs for children and adolescents. For instance, it could be useful to analyze, through longitudinal studies, the reasons why more vulnerable children have less access to dental health services. Furthermore, it is vital to carry out periodic studies into the dental health problems prevalent in children and adolescents, so that preventive action can be properly planned, such as teaching children, adolescents, and their families the benefits of following a balanced diet and improving their habits of oral hygiene, in the context of the children’s different social, economic and demographic conditions.

The present study also has some limitations. Firstly, due to the cross-sectional design, it is not possible to assign causality between the utilization of dental health services, dental health problems, and socioeconomic and demographic variables. In addition, it should be remembered that the data collected in the survey was obtained indirectly from the informants’ self-reporting, which can be affected by memory and/or social desirability bias. On the other hand, one strength of our study is that since the data were derived from a national survey, they have been obtained using carefully planned methodology, including sampling, well-designed forms, preparation of the survey participants, supervision of the survey and filtering of the data, all of which guarantee a representative sample of the population and lead to a greater understanding of these health problems and their social context.

## 5. Conclusions

The utilization of dental health services in children and young people is clearly well below the recommended level, and this is even lower in the immigrant population. The most common type of dental visit made by children is for a check-up. The probability of non-regular utilization of dental services is lower in Spanish and immigrant children from the age of 7 upwards and increases as the social class decreases. In addition, the likelihood of suffering from dental health problems is higher in both groups from the age of 7 and in the lower social class, and is lower in Spanish children living in towns with a population over 10,000.

## Figures and Tables

**Table 1:** Utilization of dental health services and oral health problems in the Spanish and immigrant child-adolescent population. Spanish National Health Survey, 2017.

Variables	Spanish (*n* = 4315) *n* (%)	Immigrants (*n* = 253) *n* (%)	*p*-Value
Time since the last visit to the dentist, stomatologist or dental hygienist			
≤12 months	2786 (64.57)	122 (48.22)	<0.001
>12 months	1529 (35.43)	131 (51.78)	
Do you have caries?			
Yes	401 (9.29)	47 (18.58)	<0.001
No	3914 (90.71)	206 (81.42)	
Do you have any teeth/molars with fillings or capped teeth?			
Yes	1093 (25.33)	62 (24.51)	0.75
No	3222 (74.67)	191 (75.49)	
Do your gums bleed spontaneously or when you brush your teeth?			
Yes	180 (4.17)	14 (5.53)	0.6
No	4135 (95.83)	239 (94.47)	

**Table 2:** Association between non-regular utilization of dental services and socioeconomic and demographic variables in Spanish and immigrant child-adolescent population. Spanish National Health Survey, 2017.

	Spanish (*n* = 4315)			Immigrants (*n* = 253)		
	*n* (%)	OR (CI 95%)	ORa (CI 95%)	*n* (%)	OR (CI 95%)	ORa (CI 95%)
Total	1529 (35.43)			131 (51.78)		
Gender						
Male	811 (53.04)	Reference	Reference	77 (58.78)	Reference	Reference
Female	718 (46.96)	0.89 (0.79–1.01)	0.94 (0.82–1.08)	54 (41.22)	0.66 (0.40–1.08)	0.60 (0.35–1.03)
Age						
3–6 years old	812 (53.11)	Reference	Reference	59 (45.04)	Reference	Reference
7–10 years old	409 (26.75)	0.18 (0.15–0.21) ^1^	0.17 (0.15–0.20) ^1^	45 (34.35)	0.35 (0.19–0.66) ^1^	0.31 (0.15–0.64) ^1^
11–14 years old	308 (20.14)	0.16 (0.14–0.20) ^1^	0.15 (0.13–0.18) ^1^	27 (20.61)	0.21 (0.11–0.42) ^1^	0.19 (0.09–0.41) ^1^
Size of town of residence						
<10,000 inhab	305 (19.95)	Reference	Reference	17 (12.98)	Reference	Reference
10,000–100,000 inhab	658 (43.03)	1.04 (0.89–1.25)	1.09 (0.91–1.32)	83 (63.36)	1.44 (0.20–2.07)	1.01 (0.36–2.79)
>100,000 inhab	566 (37.02)	1.05 (0.79–1.11)	1.11 (0.92–1.34)	31 (23.66)	1.58 (0.22–2.57)	1.89 (0.29–2.74)
Type of household						
Couple	1204 (78.74)	Reference	Reference	101 (77.10)	Reference	Reference
Father or mother	146 (9.55)	0.76 (0.62–1.94)	0.67 (0.54–1.84)	19 (14.50)	1.39 (0.65–2.97)	1.57 (0.69–3.55)
Other type of household	179 (11.71)	0.99 (0.82–1.22)	0.93 (0.75–1.15)	11 (8.40)	0.80 (0.34–1.88)	0.68 (0.28–1.68)
Social class						
Upper	316 (20.67)	Reference	Reference	8 (6.11)	Reference	Reference
Middle	491 (32.11)	1.12 (1.05–1.20) ^1^	1.16 (1.11–1.23) ^1^	21 (16.03)	1.58 (1.30–2.53) ^1^	1.13 (1.35–3.63) ^1^
Lower	722 (47.22)	1.34 (1.14–1.58) ^1^	1.59 (1.33–1.90) ^1^	102 (77.86)	1.80 (1.69–4.68) ^1^	2.42 (1.84–2.97) ^2^

OR: odds ratio; ORa: odds ratio adjusted for all socioeconomic and demographic variables; CI 95%: 95% Confidence Interval; *n*: number of people not using dental services regularly; inhab.: inhabitants; ^1^ *p* < 0.001; ^2^ *p* < 0.05. Nagelkerke’s R^2^ for Spanish model: 0.20; *p*-value for Spanish model: < 0.001. Nagelkerke’s R^2^ for immigrant model: 0.16; *p*-value for immigrant model: <0.001.

**Table 3:** Association between the presence of dental health problems and socioeconomic and demographic variables in Spanish and immigrant child-adolescent population. Spanish National Health Survey, 2017.

	Spanish (*n* = 4315)			Immigrants (*n* = 253)		
	*n* (%)	OR (CI 95%)	ORa (CI 95%)	*n* (%)	OR (CI 95%)	ORa (CI 95%)
Total	1342 (31.10)			92 (36.36)		
Gender						
Male	668 (49.78)	Reference	Reference	51 (55.43)	Reference	Reference
Female	674 (50.22)	1.08 (0.96–1.24)	1.04 (0.91–1.19)	41 (44.57)	0.89 (0.54–1.50)	0.94 (0.54–1.64)
Age						
3–6 years old	163 (12.15)	Reference	Reference	13 (14.13)	Reference	Reference
7–10 years old	527 (39.27)	2.29 (1.52–2.32) ^1^	2.27 (1.52–2.47) ^1^	35 (38.04)	2.53 (1.65–2.64) ^1^	2.36 (1.62–2.88) ^1^
11–14 years old	652 (48.58)	2.29 (1.55–2.41) ^1^	2.36 (1.57–2.53) ^1^	44 (47.83)	2.58 (1.93–2.78) ^1^	2.14 (1.62–2.30) ^1^
Size of town of residence						
<10,000 inhab	291 (21.68)	Reference	Reference	7 (7.61)	Reference	Reference
10,000–100,000 inhab	543 (40.46)	0.85 (0.52–0.97) ^2^	0.84 (0.43–0.91) ^2^	61 (66.30)	1.41 (0.56–1.57)	0.45 (0.15–1.38)
>100,000 inhab	508 (37.85)	0.79 (0.72–0.92) ^2^	0.77 (0.64–0.93) ^2^	24 (26.09)	1.93 (0.69–2.35)	0.83 (0.25–1.79)
Type of household						
Couple	1.056 (78.69)	Reference	Reference	73 (79.35)	Reference	Reference
Father or mother	145 (10.80)	0.92 (0.75–1.13)	0.98 (0.79–1.21)	13 (14.13)	1.16 (0.54–2.49)	1.12 (0.49–2.56)
Other type of household	141 (10.51)	0.86 (0.70–1.06)	0.89 (0.72–1.11)	6 (6.52)	0.57 (0.22–1.49)	0.62 (0.22–1.73)
Social class						
Upper	277 (20.64)	Reference	Reference	4 (4.35)	Reference	Reference
Middle	450 (33.53)	1.08 (0.90–1.29)	1.08 (0.90–1.29)	63 (68.48)	2.02 (0.64–2.34)	1.60 (0.48–2.33)
Lower	615 (45.83)	1.26 (1.06–1.49) ^2^	1.18 (0.99–1.41) ^2^	25 (27.17)	3.23 (1.95–4.01) ^2^	2.55 (1.70–3.31) ^2^

OR: odds ratio; ORa: odds ratio adjusted for all socioeconomic and demographic variables; CI 95%: 95% Confidence Interval; *n*: number of people with dental health problems; inhab.: inhabitants; ^1^ *p* < 0.001; ^2^ *p* < 0.05. Nagelkerke’s R^2^ for Spanish model: 0.10; *p*-value for Spanish model: <0.001. Nagelkerke’s R^2^ for immigrant model: 0.12; *p*-value for immigrant model: <0.001.

## Supplementary Materials

The following are available online at https://www.mdpi.com/1660-4601/17/3/738/s1, File S1: Research data.

## References

[1] Kassebaum N.J., Smith A.G.C., Bernabé E., Fleming T.D., Reynolds A.E., Vos T., Murray C.J.L., Marcenes W., GBD 2015 Oral Health Collaborators (2017). Global, regional, and national prevalence, incidence, and disability-adjusted life years for oral conditions for 195 countries, 1990–2015: A systematic analysis for the global burden of diseases, injuries, and risk factors. J. Dent. Res..

[2] Muñoz Pino N., Vives Cases C., Agudelo Suárez A.A., Ronda Pérez E. (2018). Comparing oral health services use in the Spanish and immigrant working population. J. Immigr. Health.

[3] Bravo M., San Martín L., Casals E., Eaton K., Widström E. (2015). The healthcare system and the provision of oral healthcare in European Union member states. Part 2: Spain. Br. Dent. J..

[4] Tchicaya A., Lorentz N. (2014). Socioeconomic inequalities in the non-use of dental care in Europe. Int. J. Equity Health.

[5] Reda S.M., Krois J., Reda S.F., Thomson W.M., Schwendicke F. (2018). The impact of demographic, health-related and social factors on dental services utilization: Systematic review and meta-analysis. J. Dent..

[6] Eurostat: Population without the Citizenship of the Reporting Country. https://ec.europa.eu/eurostat/tgm/graph.do?tab=graph&plugin=1&language=en&pcode=tps00157&toolbox=type.

[7] Reda S.F., Reda S.M., Thomson W.M., Schwendicke F. (2018). Inequality in utilization of dental services: A systematic review and meta-analysis. Am. J. Public Health.

[8] Valcárcel Soria R., Somacarrera Pérez M.L. (2016). Estado de salud oral de los niños inmigrantes en España. Odontol. Pediatr..

[9] Ghasemianpour M., Bakhshandeh S., Shirvani A., Emadi N., Samadzadeh H., Fatemi N.M., Ghasemian A. (2019). Dental caries experience and socio-economic status among Iranian children: A multilevel analysis. BMC Public Health.

[10] Schwendicke F., Dörfer C.E., Schlattmann P., Foster Page L., Thomson W.M., Paris S. (2015). Socioeconomic inequality and caries: A systematic review and meta-analysis. J. Dent. Res..

[11] Babo Soares L.F., Allen P., Bettiol S., Crocombe L. (2016). The association of socioeconomic status and dental caries experience in children in Dili, Timor-Leste. Asia Pac. J. Public Health.

[12] Kramer A.-C.A., Hakeberg M., Petzold M., Östberg A.-L. (2016). Demographic factors and dental health of Swedish children and adolescents. Acta Odontol. Scand..

[13] Peres M.A., Macpherson L.M.D., Weyant R.J., Daly B., Venturelli R., Mathur M.R., Listl S., Celeste R.K., Guarnizo Herreño C.C., Kearns C. (2019). Oral diseases: A global public health challenge. Lancet.

[14] Font Ribera L., García Continente X., Davó Blanes M.C., Ariza C., Díez E., García Calvente M.M., Maroto G., Suárez M., Rajmil L., Grupo de Determinantes Sociales de la Sociedad Española de Epidemiología (2014). El estudio de las desigualdades sociales en la salud infantil y adolescente en España. Gac. Sanit..

[15] Encuesta Nacional de Salud de España 2017. https://www.mscbs.gob.es/estadEstudios/estadisticas/encuestaNacional/encuesta2017.htm.

[16] Plan de Salud Bucodental del Ministerio de Sanidad y Política Social. Gobierno de España. http://www.mscbs.gob.es/campannas/campanas08/bucoDental/medidas_salud_bucodental.html.

[17] Domingo Salvany A., Bacigalupe A., Carrasco J.M., Espelt A., Ferrando J., Borrell C. (2014). Propuestas de clase social neoweberiana y neomarxista a partir de la Clasificación Nacional de Ocupaciones 2011. Gac. Sanit..

[18] El-Yousfi S., Jones K., White S., Marshman Z. (2019). A rapid review of barriers to oral healthcare for vulnerable people. Br. Dent. J..

[19] Markkula N., Cabieses B., Lehti V., Uphoff E., Astorga S., Stutzin F. (2018). Use of health services among international migrant children—A systematic review. Glob. Health.

[20] Ley Orgánica 4/2000, de 11 de Enero, Sobre Derechos y Libertades de los Extranjeros en España y su Integración Social. https://www.boe.es/buscar/pdf/2000/BOE-A-2000-544-consolidado.pdf.

[21] Xu M., Yuan C., Sun X., Cheng M., Xie Y., Si Y. (2018). Oral health service utilization patterns among preschool children in Beijing, China. BMC Oral Health.

[22] Tapias Ledesma M.A., Hernández Barrera V., Carrasco Garrido P., Gil de Miguel A., Esteban y Peña M., Jiménez Garcia R. (2011). Use of dental care and prevalence of caries among immigrant and Spanish-born children. ASDC J. Dent. Child..

[23] Finlayson T.L., Asgari P., Dougherty E., Tadese B.K., Stamm N., Nunez Alvarez A. (2018). Child, caregiver, and family factors associated with child dental utilization among Mexican migrant families in California. Community Dent. Health.

[24] Bhagavatula P., Xiang Q., Szabo A., Eichmiller F., Kuthy R.A., Okunseri C.E. (2012). Rural-urban differences in dental service use among children enrolled in a private dental insurance plan in Wisconsin: Analysis of administrative data. BMC Oral Health.

[25] Irvin K., Fahim F., Alshehri S., Kitsantas P. (2018). Family structure and children’s unmet health-care needs. J. Child. Health Care.

[26] Jung S.-H., Kim M.-H., Ryu J.-I. (2018). Inequalities in oral health among adolescents in Gangneung, South Korea. BMC Oral Health.

[27] Mamai Homata E., Koletsi Kounari H., Margaritis V. (2016). Gender differences in oral health status and behavior of Greek dental students: A meta-analysis of 1981, 2000, and 2010 data. J. Int. Soc. Prev. Community Dent..

[28] Kumar S., Tadakamadla J., Kroon J., Johnson N.W. (2016). Impact of parent-related factors on dental caries in the permanent dentition of 6–12-year-old children: A systematic review. J. Dent..

[29] Bright M.A., Alford S.M., Hinojosa M.S., Knapp C., Fernandez Baca D.E. (2015). Adverse childhood experiences and dental health in children and adolescents. Community Dent. Oral Epidemiol..

[30] Kumar S., Tadakamadla J., Zimmer Gembeck M.J., Kroon J., Lalloo R., Johnson N.W. (2017). Parenting practices and children’s dental caries experience: A structural equation modelling approach. Community Dent. Oral Epidemiol..

[31] Rouxel P., Chandola T. (2018). Socioeconomic and ethnic inequalities in oral health among children and adolescents living in England, Wales and Northern Ireland. Community Dent. Oral Epidemiol..

[32] Hall Scullin E., Goldthorpe J., Milsom K., Tickle M. (2015). A qualitative study of the views of adolescents on their caries risk and prevention behaviours. BMC Oral Health.

[33] Almerich Torres T., Montiel Company J.M., Bellot Arcís C., Almerich Silla J.M. (2017). Relationship between caries, body mass index and social class in Spanish children. Gac. Sanit..

[34] Kumar S., Tadakamadla J., Duraiswamy P., Kulkarni S. (2016). Dental caries and its socio-behavioral predictors—An exploratory cross-sectional study. J. Clin. Pediatr. Dent..

[35] Veiga N., Pereira C., Amaral O. (2015). Prevalence and determinants of dental caries in Portuguese children. Procedia Soc. Behav. Sci..

